# The feasibility of developing biomarkers from peripheral blood mononuclear cell RNAseq data in children with juvenile idiopathic arthritis using machine learning approaches

**DOI:** 10.1186/s13075-019-2010-z

**Published:** 2019-11-09

**Authors:** Kerry E. Poppenberg, Kaiyu Jiang, Lu Li, Yijun Sun, Hui Meng, Carol A. Wallace, Teresa Hennon, James N. Jarvis

**Affiliations:** 1 Canon Stroke and Vascular Research Center, University at Buffalo Jacobs School of Medicine & Biomedical Sciences, State University of New York, Buffalo, NY USA; 2 Department of Biomedical Engineering, University at Buffalo, State University of New York, Buffalo, NY USA; 3 Department of Pediatrics, University at Buffalo, Buffalo, NY USA; 4 Department of Computer Science and Engineering, University at Buffalo, Buffalo, NY USA; 5 Genetics, Genomics, and Bioinformatics Graduate Program, University at Buffalo, Buffalo, NY USA; 6 Department of Microbiology and Immunology, University at Buffalo, Buffalo, NY USA; 7 Department of Neurosurgery, University at Buffalo Jacobs School of Medicine & Biomedical Sciences, State University of New York, Buffalo, NY USA; 8 Department of Mechanical & Aerospace Engineering, University at Buffalo, State University of New York, Buffalo, NY USA; 9 Department of Pediatrics, University of Washington, Seattle, WA USA; 10 Pediatric Rheumatology Research, Clinical & Translational Research Center, 875 Ellicott Street, Buffalo, NY 14203 USA

**Keywords:** Juvenile idiopathic arthritis, Peripheral blood mononuclear cells, Transcriptome, Machine learning

## Abstract

**Background:**

The response to treatment for juvenile idiopathic arthritis (JIA) can be staged using clinical features. However, objective laboratory biomarkers of remission are still lacking. In this study, we used machine learning to predict JIA activity from transcriptomes from peripheral blood mononuclear cells (PBMCs). We included samples from children with Native American ancestry to determine whether the model maintained validity in an ethnically heterogeneous population.

**Methods:**

Our dataset consisted of 50 samples, 23 from children in remission and 27 from children with an active disease on therapy. Nine of these samples were from children with mixed European/Native American ancestry. We used 4 different machine learning methods to create predictive models in 2 populations: the whole dataset and then the samples from children with exclusively European ancestry.

**Results:**

In both populations, models were able to predict JIA status well, with training accuracies > 74% and testing accuracies > 78%. Performance was better in the whole dataset model. We note a high degree of overlap between genes identified in both populations. Using ingenuity pathway analysis, genes from the whole dataset associated with cell-to-cell signaling and interactions, cell morphology, organismal injury and abnormalities, and protein synthesis.

**Conclusions:**

This study demonstrates it is feasible to use machine learning in conjunction with RNA sequencing of PBMCs to predict JIA stage. Thus, developing objective biomarkers from easy to obtain clinical samples remains an achievable goal.

## Background

One of the underappreciated advances in the field of pediatric rheumatology has been the recognition that therapeutic response during the course of treated juvenile idiopathic arthritis (JIA) can be staged based on specific clinical features [1]. Using hybridization-based gene microarrays, our group has shown that these stages are associated with specific gene expression signatures in both peripheral blood mononuclear cells [2, 3] and neutrophils [3, 4]. These findings raised the possibility that gene expression patterns might be used to develop clinically useful biomarkers to guide therapeutic decision-making. For example, we have demonstrated the feasibility of using microarray-based gene expression data as a means of developing prognostic biomarkers that will determine a patient’s likelihood of achieving inactive disease within 6 months of initiating therapy [5]. However, the development of expression-based biomarkers has been hampered by the considerable inter-patient variation that is observed in gene expression studies of children with JIA, problems that are compounded when complex cell types such as peripheral blood mononuclear cells (PBMCs) are used [6].

Over the past 10 years, significant advances have been made in machine learning approaches to complex datasets. Augmented computational power and new methods have facilitated successful classification even using heterogeneous populations, such as PBMCs. For instance, Showe et al. used a support vector machine model of 29 genes expressed in PBMCs to distinguish between non-small cell lung cancer and non-malignant lung disease with 86% accuracy [7]. Serrano et al. also used support vector machines in combination with PBMC expression data to identify HIV-negative donors or HIV-positive cART-treated samples with 94% accuracy [8]. Thus, machine learning has been successful in a wide spectrum of disease classification problems.

In the present study, we renewed our efforts to determine the feasibility of developing biomarkers for disease activity in JIA from PBMCs, which are easy and relatively inexpensive to obtain and prepare from clinical blood samples. We also sought to determine whether such biomarkers might be detected in an ethnically heterogeneous population that included Native American children, as the optimal biomarkers will be those that are useful across the age range and diverse ethnicities of the JIA population. Although patient-to-patient variability in gene expression continues to be a challenge, we demonstrate that machine learning approaches can overcome this and successfully classify patients by disease activity.

## Methods

### Patients and patient characteristics

This project was reviewed and approved by the IRBs of the University of Oklahoma Health Sciences Center, the University at Buffalo, and Seattle Children’s Hospital. All research was conducted according to the IRB-approved protocol. Written informed consent documents were executed for each subject, and, where appropriate, children also executed consent documents.

Samples for this study were obtained from children with polyarticular JIA as determined from standard criteria and included three children who were rheumatoid factor positive [9], as, once again, we are seeking biomarkers that are applicable to the broadest range of children with JIA. For the purposes of this study, we examined only children with an active disease on medication with methotrexate (MTX) plus a biologic agent (ADT group) and children who had achieved clinical remission on medication (CRM group) who were on the same medications. Active disease and remission statuses were assigned according to the criteria of Wallace et al. [1]. Active disease was determined by the presence of physical signs of synovitis (warmth, synovial thickening) in at least one joint. Although the Wallace criteria assign active disease status to patients with laboratory findings that are not otherwise explained by an intercurrent infectious illness (e.g., elevated erythrocyte sedimentation rate, thrombocytosis), no patient in this study was assigned to the ADT group based on the isolated laboratory findings; all had synovitis in at least one joint. Children were assigned to the CRM group if they had no signs of active synovitis and morning stiffness lasting for < 10 min/day and no laboratory abnormalities other than those that might be attributed to a self-limited illness or medications. Furthermore, following the Wallace criteria, children in CRM had maintained this condition for six continuous months or more.

The first dataset included 12 samples from patients who were involved in a previous study [6]. Of these, 4 were from patients with ADT, while 8 were from children who fit the criteria of CRM. RNA sequencing (RNAseq) data from those samples are available on the Gene Expression Omnibus (GEO, #GSE79970). These samples were all taken from children with non-Hispanic, European ancestry.

For this study, we also performed RNAseq on 38 additional samples from children with polyarticular JIA. This study included samples from 15 patients (4 boys and 11 girls) who had achieved clinical remission on medication (CRM). Of these 15 patients, 10 achieved CRM status on methotrexate (MTX) alone, while 5 achieved CRM on MTX plus a TNF inhibitor (2 on adalimumab, 3 on etanercept). The remaining 23 (6 boys and 17 girls) were classified as having an active disease on therapy (ADT). Among the patients with ADT, 18 were on MTX alone, while 5 were on TNF inhibitors (3 on etanercept and 2 on adalimumab). There were 7 individuals from whom we collected longitudinal data; therefore, these subjects have both ADT and CRM samples. Nine samples were from individuals with mixed Native American/European ancestry recruited from the pediatric rheumatology clinic at the University of Oklahoma. Only 1 of these patients achieved CRM within a year of diagnosis, which may be a reflection of the severity of disease in the Native population, which we have previously documented [10]. In total, we analyzed an ethically heterogeneous dataset of 50 samples, 23 of which were from patients in CRM and the remaining 27 from patients in ADT.

### Cell isolation

Whole blood was drawn into 10-mL citrated CPT tubes (Becton Dickinson, Franklin Lakes, NJ). Cell separation procedures were started within 1 h from the time the specimens were drawn. Peripheral blood mononuclear cells were separated from granulocytes and red blood cells by density gradient centrifugation. Cells were then immediately placed in TRIzol® reagent (Invitrogen, Carlsbad, CA) and stored at − 80 °C.

### RNA isolation and sequencing

RNA isolation and sequencing were carried out exactly as described in [6]. In brief, total RNA was extracted using TRIzol™ reagent and further purified using the RNeasy MiniElute Cleanup kit (Qiagen, Valencia, CA). cDNA libraries were prepared for each sample using the Illumina TruSeq RNA sample preparation kit. Libraries were sequenced using 100-bp paired-end reads on the Illumina HiSeq 2500 platform.

### Data analysis

RNA sequencing data analysis was carried out using Galaxy suite of tools. Sequenced paired-end reads were aligned to the human reference genome hg19 with Bowtie2 (version 2.3.4.2) using default settings. Counts were assigned to aligned reads using htseq-count (version 0.9.1). Counts were then converted to transcripts per million (TPM) to facilitate a comparison between the samples. As data was collected during 2 different time points, we considered the 12 samples previously analyzed to be batch 1 and the 38 new samples to be batch 2. We used ComBat under default settings in R to perform batch effect correction.

We performed principal component analysis (PCA) using raw counts of all 50 samples in R using prcomp function to visually assess the presence of outliers or batch effects in our dataset.

### Model development

In order to develop and test prediction models, we randomly divided the whole cohort into training and testing cohorts. In this way, the features of the model are identified in the training cohort and then tested in an independent cohort to avoid bias. We used a 70-30 split to ensure that the testing set encompassed sufficient sample heterogeneity. In training cohort data, we employed a cutoff of average TPM > 1 and restricted our analysis to protein-coding genes to increase the likelihood that genes identified are significantly expressed. In this subset of transcripts, we performed a supervised feature selection using Hilbert-Schmidt independence criterion least absolute shrinkage and selection operator (HSIC LASSO) [11] to identify transcripts that had greatest predictive power of disease stage, i.e., ADT or CRM. This method works to find a combination of genes that consist of non-redundant features with strong dependence on disease status. Using the selected transcripts, we trained the classification models using MATLAB Statistics and Machine Learning Toolbox. Specifically, we consider four algorithms which are widely used for disease classification from expression data, including *K*-nearest neighbors (KNN) [12], random forest (RF) [13], support vector machine with cubic kernel (cSVM) [14], and SVM with Gaussian kernel (gSVM) [15]. We briefly describe each method here.

- *K*-nearest neighbors: The *K*-nearest neighbor method using a Euclidean metric was employed. The number of neighbors, *K*, was set as 10. By calculating the distance to each training sample, the testing sample was classified as the class that was most common among its *K*-nearest neighbors.
- Random forest: The random forest method was employed to build a prediction model. The number of trees was set at 1000. The random forest was built by contrasting a multitude of decision trees based on subsets of the training data generated by random sampling with replacement. The resulting model classifies testing samples by the majority vote of the decision trees.
- Support vector machine: We trained prediction models using support vector machine with two different kernels: cubic and Gaussian. To separate the binary-labeled samples, the SVM transforms them into a multidimensional space using the kernel, followed by a hyper-plane that maximizes the margin separating samples of either class. The resulting model classifies testing samples by transforming them into the higher dimensional space with the corresponding kernel and making decisions based on their signed distance to the hyper-plane.

Models were firstly evaluated using tenfold cross-validation in the training cohort. Predicted disease stage was compared to clinical diagnosis in order to determine the accuracy, sensitivity, and specificity of each model, which were averaged across the tenfold cross-validation for each model. We also implemented a receiver operating characteristic (ROC) curve analysis to determine the area under the curve (AUC) for each model as an additional performance metric. Trained models were then evaluated in an independent testing cohort. We calculated the accuracy, sensitivity, specificity, and AUC for each model based on the model predictions of testing samples.

We executed this pipeline in two different populations. First, we developed models for JIA disease stage prediction in the whole dataset. Then, since clinical experience has demonstrated that patients with Native American ancestry have more difficulty reaching remission (possibly reflecting different genetic or epigenetic influences), we sought to determine whether including this diverse patient population affected model performance. Therefore, we developed models only using samples of European descent and compared their performance to models generated using all samples.

### Pathway analysis

We used ingenuity pathway analysis (IPA) to investigate the biological pathways and networks associated with model genes using the whole dataset. Model genes with their log2-fold change of the ADT group relative to the CRM group and *t* test *p* values (both calculated using batch effect corrected TPMs of all 47 samples) were used as input. We considered disease, biological functions, and upstream regulators to be significant if the absolute value of their activation *z*-score is > 2. The *z*-score reflects the match between the observed gene expression and the predicted relationship direction. It is used to infer activation states of called functions and regulators. Networks were deemed significant if their score was greater than 15. Network score uses a hypergeometric distribution and calculated with right-tailed Fisher’s exact test in this equation: *p*-score = −log10(*p* value). This score reflects the approximate fit between the generated networks and eligible molecules from the input list.

## Results

### Data processing

For the newly sequenced samples, we achieved, on average, 73% of reads aligned concordantly exactly 1 time or more than 1 time using Bowtie2. Raw counts with a sum > 0 across all samples were used to visualize all samples by PCA. Upon visualizing the entire dataset (*n* = 50) using PCA, it became clear there were 3 outliers (603p-ADT, S17-CRM, and S17-ADT). These samples were removed prior to any model development, making our dataset 47 samples in total. We then used Combat in R to correct for batch effects present in this 47 sample dataset.

### Model development

#### Whole dataset model

**Fig. 1:**
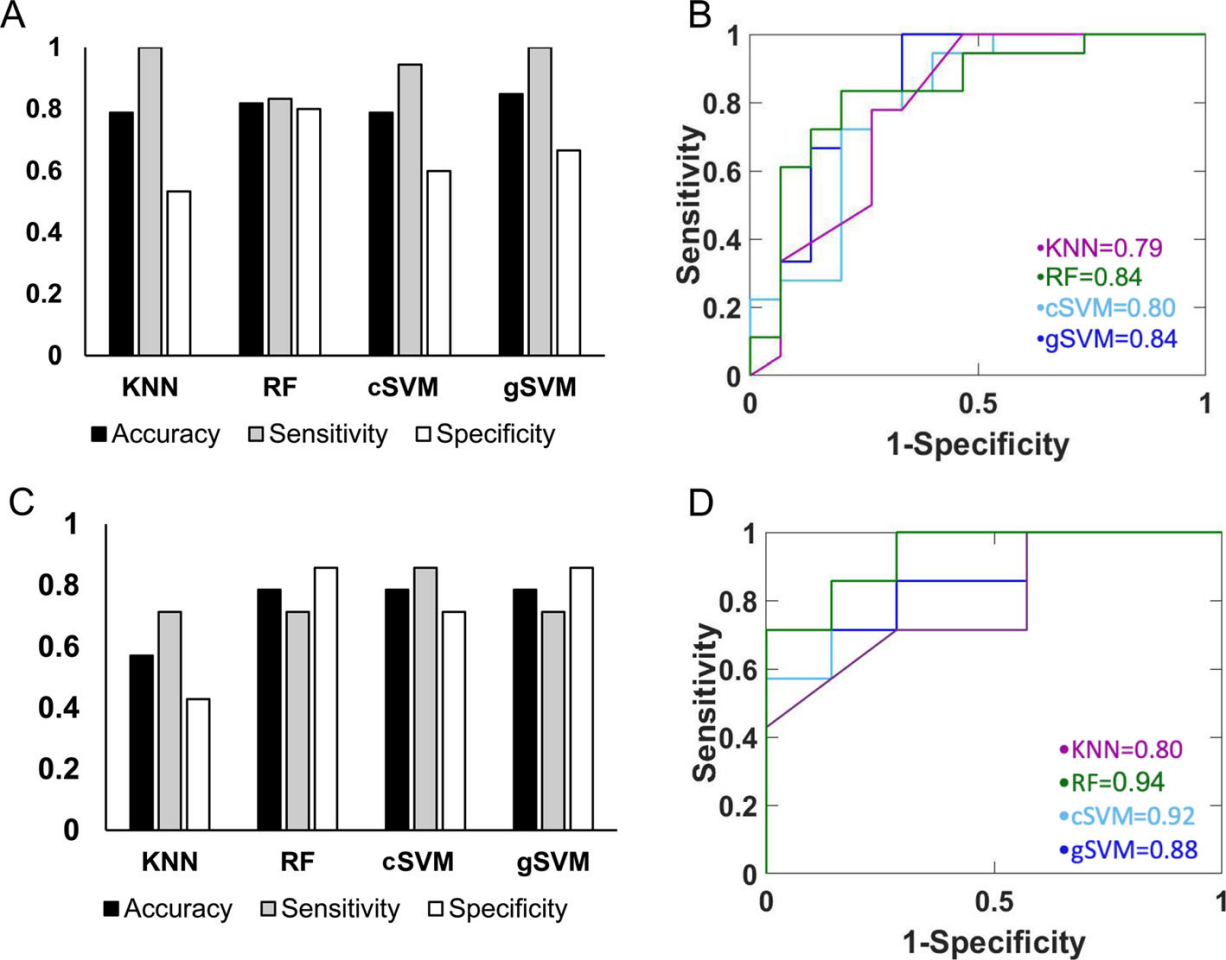
Performance of four JIA prediction models in training and testing cohorts using all samples. **a** Mean accuracy, sensitivity, and specificity for four different modeling methods (KNN, RF, cSVM, gSVM) in training as assessed by tenfold cross-validation. All had accuracies > 78%. **b** ROC analysis in the training cohort demonstrated gSVM and RF provided best classifications with an AUC of 0.84. **c** Testing accuracy, sensitivity, and specificity for four models. These are true values based on the predicted class of testing samples. RF, cSVM, and gSVM had similar performance with accuracies of approximately 79%. **d** RF had the highest AUC (0.94) of the four models tested

For the first set of models we trained, we used the entire dataset of 47 samples (25 ADT and 22 CRM). Approximately 70% of ADT and approximately 70% of CRM samples were randomly assigned to the training cohort for a total of 33 samples (18 ADT, 15 CRM). The remaining 30% of both ADT and CRM samples were used to form an independent testing cohort of 14 samples (7 ADT, 7 CRM). Cohort assignment is reported in Additional file 1: Table S1. We applied HSIC LASSO in the dataset of protein-coding transcripts with an average TPM > 1 to identify 35 genes as input for the model. The 4 models (KNN, RF, cSVM, gSVM) had accuracies > 78% in the training cohort as by tenfold cross-validation. Gaussian SVM performed the best with an AUC of 0.84. Model accuracy, sensitivity, and specificity in the training cohort are shown in Fig. 1a, while ROC curves for each model are in Fig. 1b. Models performed similarly in an independent testing cohort with accuracies > 78%, except for KNN, which only had an accuracy of 57%. Random forest outperformed other models with an AUC of 0.94. Testing accuracy, sensitivity, and specificity are shown in Fig. 1c, and ROC plots are in Fig. 1d.

#### European model

**Fig. 2:**
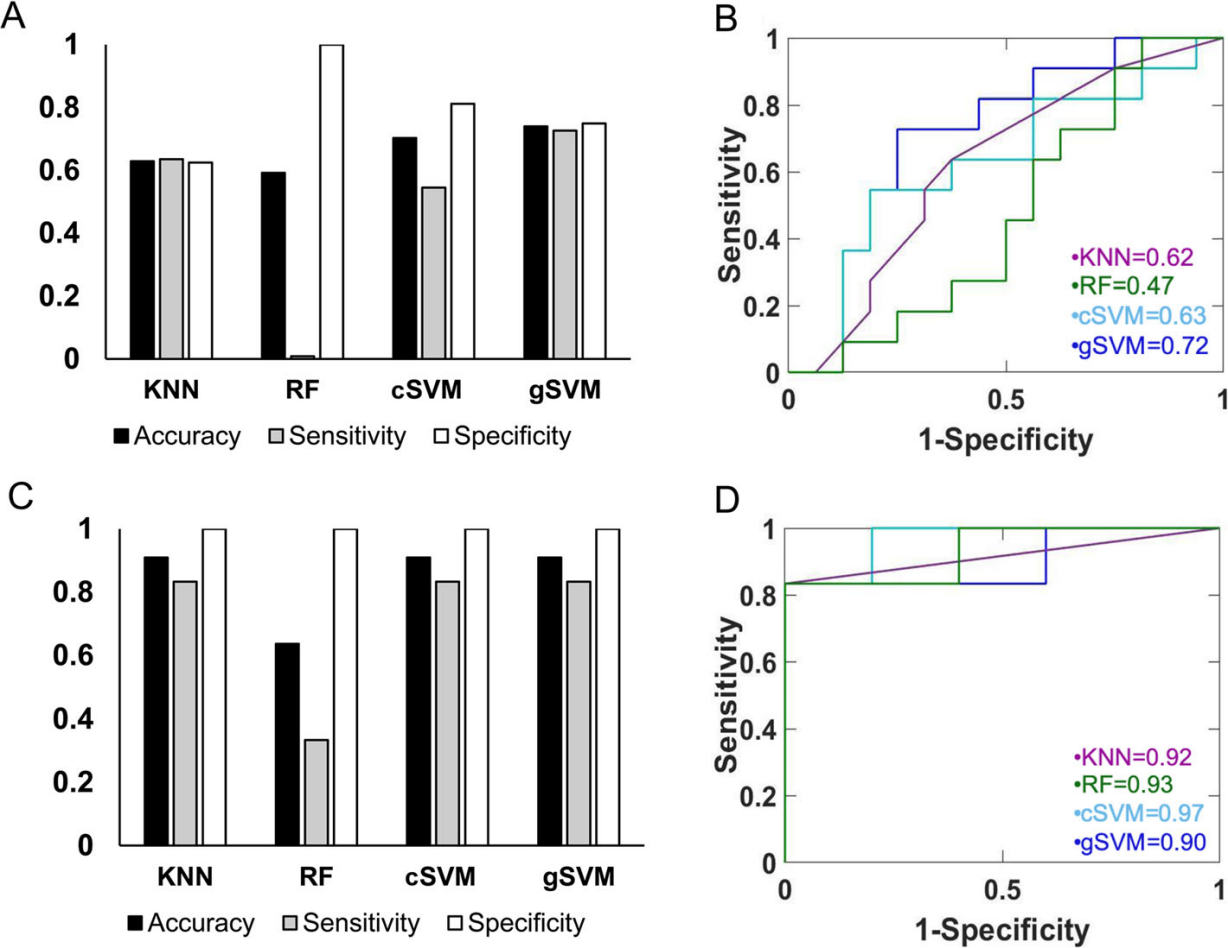
Performance of four JIA prediction models in the training and testing cohorts using only European samples. **a** Mean accuracy, sensitivity, and specificity for four models in training as assessed by tenfold cross-validation. Accuracies ranged from 59 to 74%, with gSVM having the highest accuracy. **b** Similar performance is reflected in the ROC analysis of the training cohort. gSVM again had the best performance with an AUC of 0.72. **c** Improved accuracy, sensitivity, and specificity for four models are noted in the testing cohort as assessed by true predictions of testing samples. KNN, cSVM, and gSVM all achieved a testing accuracy of 91%. **d** All models had AUCs > 0.90 in the testing cohort

The other dataset we used to develop predictive models for JIA stage consisted of all European samples. There were 38 samples in total, 17 ADT and 21 CRM. As in the whole dataset model, approximately 70% of ADT samples and approximately 70% of CRM were randomly assigned to the training cohort, which consisted of 27 samples (11 ADT, 16 CRM). The remaining samples comprised the testing cohort of 11 samples (6 ADT, 5 CRM). Cohort assignment is reported in Additional file 1: Table S1. HSIC LASSO identified 33 genes to use in model development. Gaussian SVM classified samples the best with a training accuracy of 74% and a testing accuracy of 91%. Other models’ accuracies ranged from 59 to 70% in training and 64–91% in testing. Training results and ROC plots are shown in Fig. 2a and b, while testing results and ROC plots are in Fig. 2c and d.

#### Analysis of model genes

**Fig. 3:**
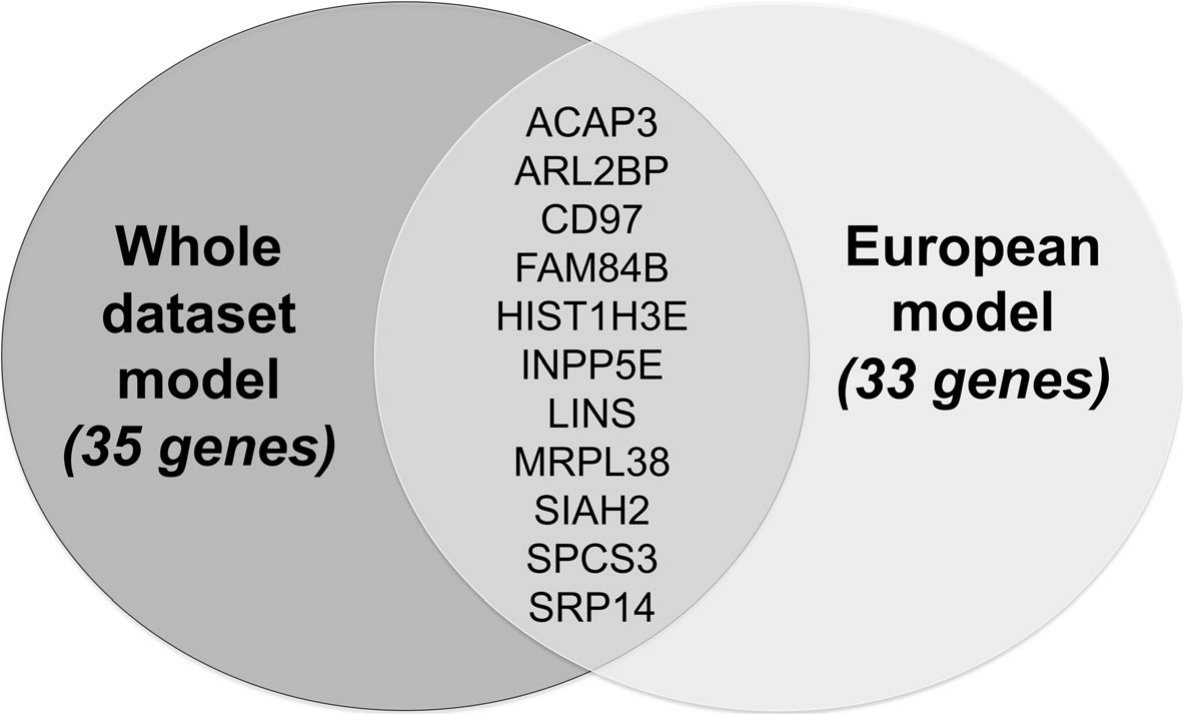
Venn diagram depicting the overlap of model genes between all sample and European sample models. Eleven genes, approximately 1/3 of the model genes, were identified by feature selection process using all samples and only European samples

Genes in each model are reported in Table 1. Those marked with an asterisk are found in both models. We note that approximately one third of the model genes were identified both in the whole dataset population and the European population. This suggests that ethnic heterogeneity does not have a dramatic effect on JIA prediction models despite different remission rates. Figure 3 depicts the overlap of classifier genes between both populations in which the models were developed.

**Table 1:** Transcripts selected by HSIC LASSO for model training using all samples and only European samples

Whole dataset model	European model
ACAP3*	AC008267.1
ARL2BP*	ACAP3*
CD97*	ARL2BP*
CEBPD	ARSA
FAM84B*	ATXN2L
GATAD1	CCDC71
HES5	CCNA2
HIST1H3E*	CD97*
IFNAR2	CKAP4
IL2RG	EPM2AIP1
INPP5E*	FAM84B*
KAT8	FANCF
KLF7	GLE1
LINS*	GSAP
MCFD2	HIST1H3E*
MID1IP1	INPP5E*
MRPL38*	KIF22
MT-CO2	L3MBTL2
MT-CYB	LINS*
MT-ND4L	MAPK8IP1
NSMAF	MRP63
PAQR7	MRPL38*
PNPLA2	NME3
PSME2	OSMR
RPL23	PPM1K
S100P	RANBP6
SIAH2*	RLTPR
SPCS3*	SIAH2*
SRP14*	SPCS3*
SSNA1	SRP14*
TCTA	TRIP13
THAP1	TXNL4B
UROD	USP51
ZAP70	
ZC3H12A	

Transcripts with an asterisk are identified in both models

We note that multiple genes within the two models are associated with biological functions that are relevant to JIA. For example, CD97 is an early activation marker on T cells and interacts with CD55 to serve as a co-stimulator [16]. CD97 also serves as an adhesion receptor on inflammatory cells and stimulates angiogenesis through binding integrin counter-receptors on endothelial cells [17]. The CCAAT/enhancer-binding protein delta (CEBPD, C/EBPδ) is a transcription factor that modulates multiple biological processes, including inflammation [18]. ZAP70 kinase is an essential protein for T cell signaling, and its absence leads to severe immunodeficiency [19]. IFNAR2 is the high-affinity receptor for interferons α and β, important modulators of innate and adaptive immunity. We provide a complete list of the genes that emerged from both models in Additional file 2: Table S2.

**Fig. 4:**
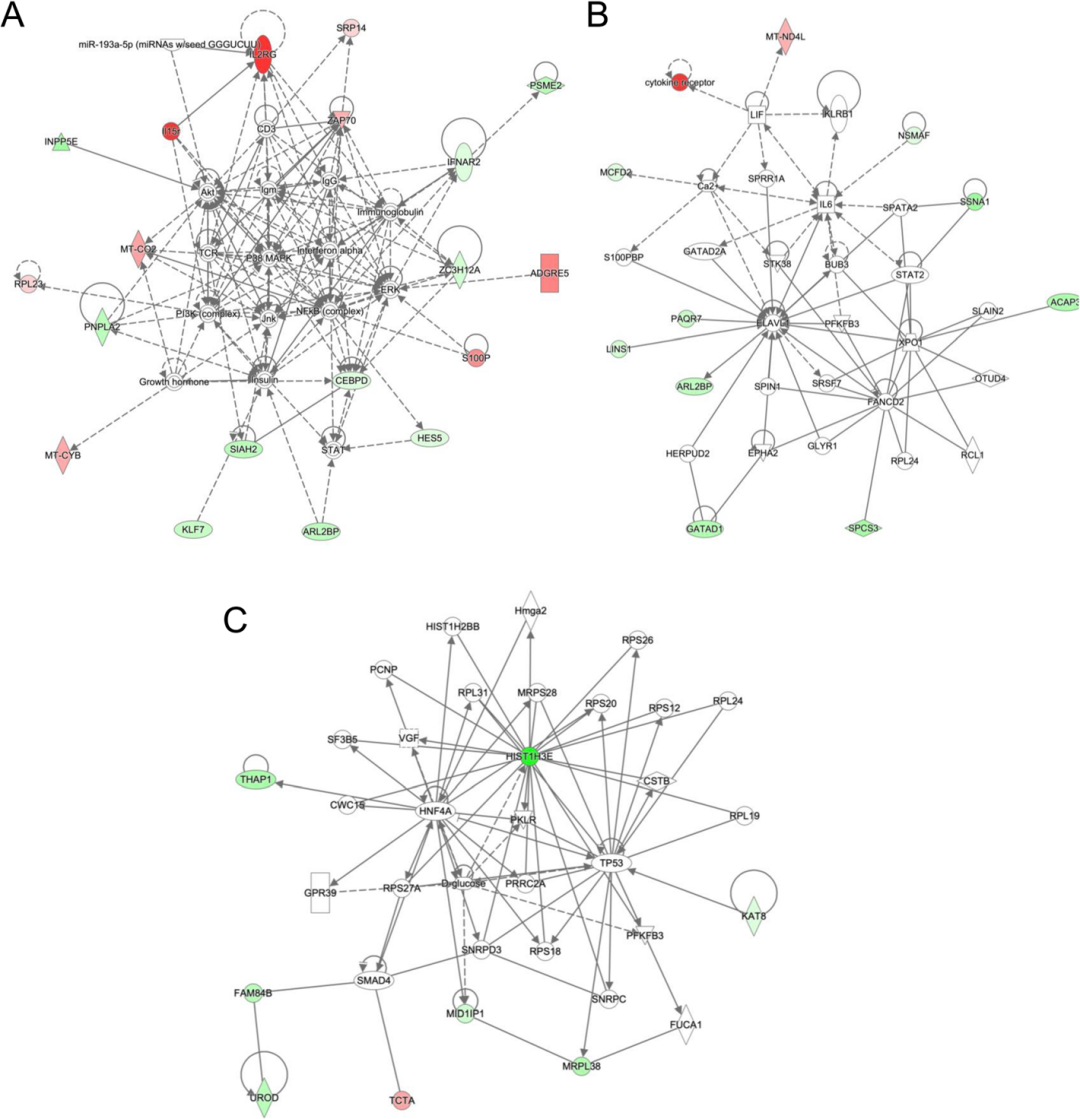
IPA networks using model genes from whole dataset analysis. Three networks were significant. Transcripts with increased expression in the ADT group are red, while transcripts with decreased expression in the ADT group are green. The color intensity represents fold change. **a** The first network (score = 46) associated with cancer, dermatological diseases and conditions, and hematological disease. **b** The second network (score = 22) associated with cell-to-cell signaling and interaction, cell morphology, and organismal injury. **c** The last network (score = 17) associated with protein synthesis, RNA damage and repair, and cancer

Due to the high degree of overlap between the model genes identified using the whole dataset or only European samples and the fact that the whole dataset model uses both more genes and more patient samples, we conducted an IPA analysis on the genes selected in the whole dataset model. Organismal death and morbidity or mortality were predicted to be increased in the ADT group (*z*-scores 2.7, 2.9). No upstream regulators were found to be significant in our data. Three networks had significant scores. The first network, which scored 46, is associated with cancer, dermatological diseases and conditions, and hematological disease. The first network, shown in Fig. 4a, included members of important inflammatory signaling such as NFKB, P38 MAPK, and ERK. The top diseases and functions of the next network, with a score of 22, were cell-to-cell signaling and interaction, cell morphology, and organismal injury and abnormalities. The last network, with a score of 17, associated with protein synthesis, RNA damage and repair, and cancer. Networks 2 and 3 are shown in Fig. 4b and c. Thus, the models developed with the machine learning process incorporated not only select, biologically relevant genes, but were also genes that, as a group, reflected biologically coherent processes.

## Discussion

Numerous studies have documented differences in the gene expression profiles in PBMC when children with JIA are compared to healthy children [20]. The specific differences in the expression profiles from what is seen in healthy children vary from one JIA subtype to another [21]. Thus, there has been considerable interest in exploiting these features of JIA both to develop clinically useful assays (e.g., for diagnosis and prognosis) and to better understand the biology of treatment response [22].

As the attainment of remission has now become a reachable goal in both pediatric rheumatology clinical trials and the “target” of “treat-to-target” therapeutic strategies [23], there is a pressing need for objective biomarkers that will confirm that remission has been achieved. We have shown that treatment response in JIA is a highly complex, non-linear process associated with considerable re-ordering of peripheral blood cell transcriptomes after treatment is initiated [24, 25]. The resulting state of “remission,” as reflected in the peripheral blood transcriptomes, is not a “normalization” of expression patterns, but, rather, a new and distinct state that differs little whether remission (CRM) was achieved using methotrexate alone or methotrexate combined with a TNF inhibitor [3]. These findings suggested that peripheral PBMC expression signatures might be used to determine objectively that an individual patient had achieved remission, regardless of the medications used.

Our earliest attempts in developing expression-based biomarkers from PBMC expression data were hindered by both the heterogeneity of the cell population and the heterogeneity of the patients [26]. However, since that earlier paper, numerous studies have demonstrated the utility of machine learning analytic approaches in developing biomarkers for disease “states,” even when heterogeneous cell and patient populations are studied [7, 8, 27–29]. For example, we have recently demonstrated that machine learning analysis of peripheral blood neutrophil expression data can accurately identify patients with intracranial aneurysms [30, 31]. We have therefore renewed our efforts to use PBMC expression data to develop biomarkers of active disease and remission in polyarticular JIA.

In the current study, we demonstrate that is it possible to predict the JIA disease stage, whether active disease or remission (CRM), using PBMC expression data and machine learning methods. Furthermore, we demonstrate that the models performed equally well whether we included only non-Hispanic, European-descended subjects or a larger cohort that included enrolled members of Native American tribes from Oklahoma. Indeed, the models performed better when the Native American patients were added, although this may simply have been due to the fact that adding these patients increased the number of subjects and thus the accuracy and power of the predictive models. We expect that we will further improve the predictive power by further increasing the sample size. Implementing other machine learning methods, such as neural networks [32], may further improve our ability to classify samples from a heterogeneous cell population.

Our results were corroborated by functional analysis of the genes that were used to discriminate active disease and remission. The discriminating transcripts included genes plausibly linked to the biology of therapeutic response (a process about which we still understand very little). Furthermore, the functional associations with MAP kinase signaling, intercellular communication, and cell adhesion are consistent with the findings from our previous gene expression studies [2, 3, 5].

We were unable to discriminate between children who achieved remission on MTX alone vs. those who achieved remission on MTX plus a biological agent. These findings are consistent with our earlier paper showing the CRM, as defined by the Wallace criteria, represents a distinct immunologic/transcriptional state that differs a little (at least in PBMC) whether that state was achieved with MTX or MTX plus a biological agent [3]. These findings suggest that there are specific immune “set points” that must be reached in order for the remission state to be achieved. Our findings from neutrophils are also consistent with that hypothesis [25].

Despite these promising results, we are still faced with the challenge of using PBMC expression data from individual patients to assign “active disease” or “CRM” status, which will be required if such information is to be used to inform clinical care. Achieving this goal will require several additional steps, which will include [1] refining this model using a larger cohort of patients [2], validating the model on an independent cohort of patients, and [3] blindly assessing a third cohort to assign status in individual expression profiles. These are all achievable goals. Furthermore, it seems likely that the problem of using a bulk population of heterogeneous cells (PBMC) might be overcome using single-cell RNAseq methods. While this approach comes with its own analytic challenges [33], there is no reason, a priori, that machine learning approaches could not be adapted to these types of datasets to developed classification models. Furthermore, single-cell data may provide useful insights into the mechanisms leading to the attainment of remission that cannot be elucidated using bulk populations of heterogeneous cells.

Another step that will need to be taken before expression-based assessments of CRM come into clinical use will be the demonstration that this approach is superior than the method currently in use: application of the clinical and laboratory findings that make up the Wallace criteria (1). There is an ongoing concern in the field that physical examination alone is insensitive to detect subtle, smoldering synovitis, for example [34]. In the present study, we limited ourselves to making a head-to-head comparison between expression profiling and the existing “gold standard,” i.e., the Wallace criteria. However, future studies will invariably require an assessment of the reliability of expression profiling vs. the Wallace criteria at, for example, predicting disease flares on or off medication.

## Conclusions

PBMCs remain a promising source for the development of expression-based biomarkers, provided that the proper analytic tools are used to develop classification algorithms. The ease with which these cells can be obtained and the continued improvement and affordability of sequencing techniques make this a fruitful line of research despite the inherent challenges of cell and patient heterogeneity.

## Supplementary information

**Additional file 1: Table S1.** Sample information.

**Additional file 2: Table S2.** Gene names and functions.

## Data Availability

The datasets generated and/or analyzed during the current study are available in the GEO repository under GSE79970 and GSE79970.

## Abbreviations

ADT: Active disease on therapy
AUC: Area under the curve
CRM: Clinical remission on medication
cSVM: Support vector machine with a cubic kernel
gSVM: Support vector machine with a Gaussian kernel
HSIC: Hilbert-Schmidt independence criterion
IPA: Ingenuity pathway analysis
JIA: Juvenile idiopathic arthritis
KNN: *K*-nearest neighbor
LASSO: Least absolute shrinkage and selection operator
PBMC: Peripheral blood mononuclear cell
PCA: Principal component analysis
RF: Random forest
RNASeq: RNA sequencing
ROC: Receiver operating characteristic curve
TPM: Transcripts per million
