# Supplementary material for: The feasibility of developing biomarkers from peripheral blood mononuclear cell RNAseq data in children with juvenile idiopathic arthritis using machine learning approaches
Source: Arthritis Res Ther. 2019 Nov 9;21:230. doi: 10.1186/s13075-019-2010-z (PMC6842535; doi:10.1186/s13075-019-2010-z)
Supplement: Supplementary file 1 — Additional file 1: Table S1. Sample information. [file 13075_2019_2010_MOESM1_ESM.docx]

Additional file 1: **Table S1.** Sample information.

| **Sample ID** | **Class** | **Batch** | **Whole dataset model cohort** | **Ancestry** | **European model cohort** |
| --- | --- | --- | --- | --- | --- |
| 246p-ADT | ADT | Batch 1 | Training | European | Training |
| 584P-ADT | ADT | Batch 1 | Testing | European | Training |
| 677p-ADT | ADT | Batch 1 | Training | European | Testing |
| 684p-ADT | ADT | Batch 1 | Training | European | Training |
| 698p-ADT | ADT | Batch 1 | Training | European | Training |
| 719p-ADT | ADT | Batch 1 | Training | European | Testing |
| 729p-ADT | ADT | Batch 1 | Testing | European | Training |
| 828p-ADT | ADT | Batch 1 | Testing | European | Training |
| 892p-ADT | ADT | Batch 1 | Training | European | Training |
| S30-ADT-P | ADT | Batch 1 | Training | European | Training |
| S41-ADT-P | ADT | Batch 1 | Testing | European | Training |
| S45-ADT-P | ADT | Batch 1 | Training | European | Training |
| S7-ADT-P | ADT | Batch 1 | Testing | European | Testing |
| ADT1027 | ADT | Batch 2 | Training | European | Testing |
| ADT951 | ADT | Batch 2 | Training | European | Training |
| ADT874 | ADT | Batch 2 | Training | European | Testing |
| ADT928 | ADT | Batch 2 | Training | European | Testing |
| 1112P-ADT | ADT | Batch 1 | Training | Mixed | - |
| 1114p-ADT | ADT | Batch 1 | Training | Mixed | - |
| 477p-ADT | ADT | Batch 1 | Testing | Mixed | - |
| 739p-ADT | ADT | Batch 1 | Training | Mixed | - |
| 761p-ADT | ADT | Batch 1 | Training | Mixed | - |
| 824p-ADT | ADT | Batch 1 | Training | Mixed | - |
| 952p-ADT | ADT | Batch 1 | Testing | Mixed | - |
| 995p-ADT | ADT | Batch 1 | Training | Mixed | - |
| 1005p-CRM | CRM | Batch 1 | Training | European | Training |
| 1086p-CRM | CRM | Batch 1 | Testing | European | Training |
| 1121p-CRM | CRM | Batch 1 | Training | European | Training |
| 372p-CRM | CRM | Batch 1 | Training | European | Training |
| 847p-CRM | CRM | Batch 1 | Training | European | Training |
| 900P-CRM | CRM | Batch 1 | Training | European | Training |
| 904p-CRM | CRM | Batch 1 | Testing | European | Testing |
| 950p-CRM | CRM | Batch 1 | Testing | European | Testing |
| S30-CRM-P | CRM | Batch 1 | Testing | European | Training |
| S41-CRM-P | CRM | Batch 1 | Training | European | Testing |
| S45-CRM-P | CRM | Batch 1 | Training | European | Training |
| S7-CRM-P | CRM | Batch 1 | Testing | European | Training |
| 725P-CRM | CRM | Batch 1 | Training | European | Training |
| CRM1119 | CRM | Batch 2 | Training | European | Training |
| CRM1116 | CRM | Batch 2 | Testing | European | Training |
| CRM612 | CRM | Batch 2 | Training | European | Training |
| CRM592 | CRM | Batch 2 | Training | European | Training |
| CRM931 | CRM | Batch 2 | Testing | European | Training |
| CRM848 | CRM | Batch 2 | Training | European | Testing |
| CRM799 | CRM | Batch 2 | Training | European | Training |
| CRM642 | CRM | Batch 2 | Training | European | Testing |
| 926p-CRM | CRM | Batch 1 | Training | Mixed | - |

“Mixed” in ancestry column refers to mixed European/Nativ
