# Supplementary material for: The feasibility of developing biomarkers from peripheral blood mononuclear cell RNAseq data in children with juvenile idiopathic arthritis using machine learning approaches
Source: Arthritis Res Ther. 2019 Nov 9;21:230. doi: 10.1186/s13075-019-2010-z (PMC6842535; doi:10.1186/s13075-019-2010-z)
Supplement: Supplementary file 2 — Additional file 2: Table S2. Gene names and functions. [file 13075_2019_2010_MOESM2_ESM.docx]

Additional file 2: **Table S2.** Gene Names and Functions.

*Genes identified in both models*

Gene ID Gene Name Known/Putative Biological Function

ACAP3 ArfGAP With Coiled-Coil, Ankyrin Repeat And PH Domains 3 Cellular GTPase

## ARL2BP ADP ribosylation factor like GTPase 2 binding protein Ras-related GTPase

## CD97  Adhesion G protein-coupled receptor E5 Adhesion molecule

FAM84B Family With Sequence Similarity 84 Member B Function unknown. Known to interact with alpha-1 catenin

HIST1H3E Histone cluster 1, H3e Histone protein

## INPP5E Inositol polyphosphate-5-phosphatase E Mobilizes intracellular calcium and acts as a second messenger

LINS Lines homologue 1 WNT signaling protein

MRPL38 Mitochondrial ribosomal protein L38 Protein component of human riobosomes

SIAH2 Siah E3 Ubiquitin Protein Ligase 2 E3 ligase involved in protein ubiquitination and degradation

SPCS3 Signal peptidase complex S3 Targets proteins to the ER

SRP14 Signal recognition particle 14 Targets secretory proteins to the rough ER membrane

*Genes identified only in the whole data set model*

CEBPD CCAAT Enhancer Binding Protein Delta Transcription factor

GATAD1 GATA Zinc Finger Domain Containing 1 Binds to a histone modification site to regulate transcription

HES5 Hes Family BHLH Transcription Factor 5 Transcriptional repressor

IFNAR2 Interferon Alpha And Beta Receptor Subunit 2 One of the 2 chains for the receptor for interferons alpha and beta

IL2RA Interleukin 2 receptor subunit gamma Common gamma chain for multiple cytokine receptors

KAT8 Lysine acetyl transferase 8 Histone modifying enzyme

KLF7 Krueppel-like factor 7 Transcriptional regulator

MCFD2 Multiple Coagulation Factor Deficiency 2 Assists in transporting coagulation factors from the ER to the Golgi

MID1IP1 Mid1-interacting protein 1 Regulates lipogenesis in the liver

MT-CO2 Mitochondrially Encoded Cytochrome C Oxidase II (COX2) Regulator of inflammation through generation of eicosanoids

MT-CYB Mitochondrially Encoded Cytochrome B Involved in oxidative phosphorylation

MT-ND4L Mitochondrially encoded NADH:ubiquinone Involved in ATP production

oxidoreductase core subunit 4L

NSMF Neutral Sphingomyelinase Activation Associated Factor May play a role in TNF-mediated immune/inflammatory responses

PAQR7 Progestin And AdipoQ Receptor Family Member 7 May regulate steroid hormone receptor activation

## PNPLA2 Patatin like phospholipase domain containing 2 Involved in triglyceride metabolism

PSME2 Proteasome activator subunit 2 Involved in processing MHC1 peptides

RPL23 Ribosomal Protein L23 Ribosomal structural protein

S100P S100 Calcium Binding Protein P Calcium binding protein; may be involved in cell cycle progression and

differentiation

**Table S2.** Gene Names and Functions (cont’d)

*Genes identified only in the whole data set model (cont’d)*

Gene ID Gene Name Known/Putative Biological Function

SSNA1 Sjogren's Syndrome Nuclear Autoantigen 1 Cell cycle regulator

TCTA T Cell Leukemia Translocation Altered Little functional data

THAP1 THAP Domain Containing 1 Transcription factor

UROD Uroporphyrinogen Decarboxylase Involved in heme biosynthesis

ZAP70 Zeta Chain Of T Cell Receptor Associated Protein Kinase 70 Important for T cell development and activation

ZC3H12A Monocyte Chemotactic Protein-Induced Protein 1 Multi-faceted regulator of inflammation/immune activation

*Genes identified only in the European model*

AC008267.1 Guanine Nucleotide Binding Protein (G Protein) Non-coding RNA

ARSA Arylsulfatase A Sulfatide processing enzyme

ATXN2L Ataxin 2 Like Ataxin type 2 related protein of unknown function

CCDC71 Coiled-Coil Domain Containing 71 No functional information in public databases

CCNA2 Cyclin A2 Cell cycle regulator

CKAP4 Cytoskeleton-Associated protein 4 Cell membrane protein. May play a role in signal transduction.

EPM2AIP1 EPM2A Interacting Protein 1 Function unknown

FANCF Fanconi Anemia Complementation Group F DNA repair protein

GLE1 GLE1 RNA Export Mediator Required for the export of poly-A RNA from the nucleus to the cytoplasm

GSAP Gamma-Secretase Activating Protein Catalyzes the formation of beta amyloid

KIF22 Kinesin Family Member 22  Kinesin-dependent molecular motor

L3MBTL2 L3MBTL Histone Methyl-Lysine Binding Protein 2 Maintains repressive chromatin state

MAPK8IP1 Mitogen-Activated Protein Kinase 8 Interacting Protein 1 Inhibits MAP kinase function

MRP63 Mitochondrial Ribosomal Protein 63 Ribosomal subunit protein

NME3 NME/NM23 Nucleoside Diphosphate Kinase 3 Catalyzes nucleoside triphosphates other than ATP. May inhibit granulocyte

differentiation.

OSMR Oncostatin M/ IL-31 receptor Regulator of hematopoiesis and inflammation

PPM1K Protein Phosphatase, Mg2+/Mn2+ Dependent 1K Mitochondrial protein

RANBP6 RAN Binding Protein 6  Nuclear transport receptor

RLTPR Capping Protein Regulator And Myosin 1 Linker 2 Scaffolding protein required for CD28 co-stimulation of T cells

TRIP13 Thyroid Hormone Receptor Interactor 13 Regulator of cell division

TXNL4B Thioredoxin Like 4B  Regulates cell cycle progression and RNA splicing

USP51 Ubiquitin Specific Peptidase 51 Regulates DNA damage response
